# Nephrology workforce in China: describing current status and evaluating the optimal capacity based on real-world data

**DOI:** 10.1186/s12960-023-00851-3

**Published:** 2023-08-08

**Authors:** Jingyi Wu, Qing Li, Chenlu Bao, Chao Yang, Pengfei Li, Luxia Zhang

**Affiliations:** 1 Advanced Institute of Information Technology, Peking University, No.233 Yonghui Road, Xiaoshan District, Hangzhou, 311215 Zhejiang China; 2 Renal Division, Department of Medicine, Peking University First Hospital, Peking University Institute of Nephrology, Beijing, 100034 China; 3 National Institute of Health Data Science, Peking University, No.38 Xueyuan Road, Haidian District, Beijing, 100191 China

**Keywords:** Nephrology workforce, Workforce capacity, Patient mobility, Health policy

## Abstract

**Objective:**

This study aims to characterize the current status of the nephrology workforce in China and evaluate its optimal capacity based on real-world patient mobility data.

**Methods:**

Data on nephrologists in China were collected from two prominent online healthcare platforms using web crawlers and natural language processing techniques. Hospitalization records of patients with chronic kidney disease (CKD) from January 2014 to December 2018 were extracted from a national administrative database in China. City-level paths of patient mobility were identified. Effects of nephrology workforce on patient mobility were analyzed using multivariate Poisson regression models.

**Results:**

Altogether 9.13 nephrologists per million population (pmp) were in practice, with substantial city-level variations ranging from 0.16 to 88.79. The ratio of nephrologists to the estimated CKD population was 84.57 pmp. Among 6 415 559 hospitalizations of patients with CKD, 21.3% were cross-city hospitalizations and 7441 city-level paths of patient mobility with more than five hospitalizations were identified. After making adjustment for healthcare capacity, healthcare insurance, economic status, and travel characteristics, the Poisson regression models revealed that the number of nephrologists in both the source city (incidence rate ratio [IRR] 0.99, per 1 pmp increase) and destination city (IRR 1.07, per 1 pmp increase) were independently associated with patient mobility. An IRR plateau was observed when the number of nephrologists exceeded 12 pmp in the source city, while a rapidly increasing IRR was observed beyond 20 pmp in the destination city.

**Conclusions:**

The nephrology workforce in China exhibits significant geographic variations. Based on local healthcare needs, an optimal range of 12–20 nephrologists pmp is suggested.

**Supplementary Information:**

The online version contains supplementary material available at 10.1186/s12960-023-00851-3.

## Introduction

Chronic kidney disease (CKD) is increasingly being acknowledged as a significant global public health concern due to its association with a rising risk of numerous life-threatening diseases [1]. Estimates indicate a global prevalence of CKD of 9.4–13.4%, and CKD rose to become the 11th leading cause of death in 2017, up from 19th in 1990 [2, 3]. Furthermore, CKD is preventable and treatable, highlighting the need for it to be prioritized in global health policy-making [4]. Previous global surveys have revealed critical shortages in the workforce capacity for patients with kidney failure, with marked variations across income levels and regions [5, 6]. Although the nephrology workforce has been quantitatively described through questionnaire surveys by the American Society of Nephrology (ASN), European Renal Association–European Dialysis and Transplant Association (ERA-EDTA), and International Society of Nephrology (ISN) [6, 7], determining the optimal capacity of the nephrology workforce, as gauged by the ratio of nephrologists to the general population, remains a challenge.

Patient mobility, defined as patients seeking healthcare services outside their place of residence [8], is a phenomenon that has increased in prevalence both internationally and domestically [9]. It is widely recognized that patient mobility serves as a proxy for evaluating the quality and accessibility of hospital services, particularly in decentralized healthcare systems [10]. Additionally, structural forces within the healthcare system such as healthcare service allocation, healthcare delivery organization, and insurance, combined with an individual’s ability to travel (influenced by factors such as socioeconomic status, health status, and social support), collectively impact patient mobility [11]. Recent advancements in healthcare digitalization and the accumulation of electronic medical records have enabled researchers to investigate patient mobility, including its implications for healthcare governance [12]. However, studies focusing on kidney disease remain scarce.

In China, the estimated prevalence of CKD in adults stands at 10.8%, affecting more than 120 million individuals [13]. Over recent decades, the disease burden attributable to CKD has surged in China due to the rising prevalence of diabetes mellitus, hypertension, and obesity, and the aging of the population [14]. Despite this, the precise size and distribution of the nephrology workforce in mainland China remain elusive due to a dearth of studies and the absence of a centralized registration system [15]. The ubiquity of hospital websites with detailed staff information, and the increase in online healthcare services, in China have made it feasible to acquire information on the nephrology workforce using information technologies. Additionally, national administrative data have been employed to delve into the details of patient mobility [16, 17].

Therefore, this study aims to evaluate the optimal capacity of the nephrology workforce, by utilizing information on nephrologists acquired from the web, and employing patient mobility as a proxy indicator of optimal capacity.

## Methods

### Study area

China, an East Asian country, boasts a population exceeding 1.4 billion and has an area of nearly 9.6 million square kilometers. The administrative structure in China is divided into three levels: provincial, city, and township. The country comprises 34 provinces, with mainland China consisting of 31 provinces. In China, healthcare delivery is hierarchically structured to mirror the administrative system. This structure assumes that patients initially utilize local primary care and, if necessary, seek specialized healthcare in higher-tier cities through cross-city mobility, as higher-level hospitals tend to be concentrated in these cities [12]. Typically, patients are inclined to seek cross-city healthcare in the provincial capital when local services are deemed inadequate or when a broader range of healthcare options is sought. Prominent hospitals in cities such as Beijing and Shanghai attract patients from other provinces willing to travel considerable distances for specialized healthcare. During the study period (2014–2018), three government-led health insurance schemes were operational in China: Urban Employees’ Basic Medical Insurance (UEBMI) for city employees, Urban Residents’ Basic Medical Insurance (URBMI) for non-working urban population, and New Rural Cooperative Medical Scheme (NRCMS) for all rural residents [18]. The UEBMI and URBMI are collectively referred to as Urban Basic Medical Insurance (UBMI).

### Data sources

Data regarding the nephrology workforce in China were procured from two leading online healthcare platforms, Haodaifu (“Good Doctor” in English; www.haodf.com) and WeDoctor (www.guahao.com). Haodaifu has aggregated information on > 890 000 physicians from over 10 000 hospitals, while WeDoctor has information on 240 000 physicians from over 7200 hospitals, spanning 311 cities in China. Leveraging the exhaustive information available on these online healthcare platforms, several studies have explored healthcare practices in China [19, 20]. These platforms amass extensive data on physicians sourced from official hospital websites, encompassing location, seniority, medical specialty, and affiliated hospital. The data are regularly updated. We employed web crawlers and natural language processing techniques to extract data on all nephrologists listed on these platforms and subsequently calculated the number and proportion of nephrologists relative to physicians for each city (for more details, see Additional file 1: Text S1) [6, 21]. Notably, in China, the majority of nephrologists are hospital-based, as opposed to practicing in office-based settings, thereby minimizing the influence of office-based nephrologists on our workforce estimations. Data on nephrologists from 1899 tertiary hospitals, which account for 63.4% of all tertiary hospitals in China and span 311 cities, were extracted in this study. We performed manual data validation using information from 10 urban hospitals in four developed cities (Beijing, Shanghai, Guangzhou, and Shenzhen) and 5 hospitals in five rural regions. The mean absolute error in the number of nephrologists was 2 for urban hospitals and 1 for rural hospitals, which attests to the accuracy of the data obtained from the two platforms (for more details, see Additional file 1: Text S2).

City-level patient mobility data were aggregated from individual-level healthcare utilization information obtained from the Hospital Quality Monitoring System (HQMS). The HQMS is a mandatory patient-level national database for hospital accreditation overseen by the National Health Commission of the People’s Republic of China. HQMS collected patient-level data from the nationally uniform front page of the hospitalization medical record. As a part of stringent standard practice in China, the front page had legal validity and must be completed by the caregiving doctors who have the most accurate and comprehensive understanding of the patient’s medical condition. Moreover, the HQMS data reporting system performs automated data quality control daily at the time of data submission to ensure the completeness, consistency, and accuracy of data. Details of HQMS were provided in earlier studies [14, 22]. Briefly, the HQMS database collects standardized electronic inpatient discharge records from > 75% of tertiary hospitals across 31 provinces and 359 cities in China. Tertiary hospitals in China can provide primary, secondary, and tertiary care simultaneously and cater to a nationwide patient population. In 2019, approximately 54% of all hospital inpatients in China were tertiary hospital patients. With support from the World Health Organization (China–World Health Organization Biennial Collaborative Projects 2018–2019), we accessed the HQMS database with the primary objective of supplying evidence for policy-making concerning kidney diseases.

We employed the International Classification of Diseases-10 (ICD-10) discharge diagnoses to identify patients with CKD, some of whom experienced multiple hospitalizations. Hospitalizations were categorized as CKD if patients had at least one of the following diagnoses: diabetic nephropathy, hypertensive nephropathy, glomerulonephritis, tubulointerstitial nephritis, obstructive nephropathy, and other related diagnoses (ICD-10 codes are provided in Additional file 1: Table S1) [23]. Cross-city hospitalizations of CKD patients were indicated by the places of residence or work (source city) being different from the hospitalization location (destination city). The source city was determined using patients’ residential addresses, postcodes, and phone numbers. Between January 2014 and December 2018, 6 415 559 hospitalizations involved patients with CKD, of which 1 367 471 (21.3%) were classified as cross-city hospitalizations in this study. From these records, we identified 26 082 directed city-level paths of patient mobility within the CKD population. To ensure data representativeness, only city-level paths with more than five hospitalizations were included. Consequently, a total of 1 332 189 cross-city hospitalizations comprised 7441 city-level mobility paths, which are the basic units of regression analyses.

Data pertaining to city characteristics were also gathered to adjust for covariates, including healthcare capacity, health insurance, economic status, and transportation infrastructure [8, 24, 25]. The majority of the covariate data were sourced from the *China City Statistical Yearbook* [26]. Healthcare capacity in a city was defined by the number of hospital beds and number of physicians. The UBMI coverage proportion was utilized to gauge the status of health insurance in the source city. Average annual population and gross domestic product (GDP) per capita were used to evaluate economic status. The traffic eigenvector centrality of both source and destination cities was utilized to depict the development of transportation networks, while the highway distance between cities served as a measure of transportation convenience. Traffic data were extracted from a comprehensive transportation network, with vertices representing cities and edges representing direct flights or train connections between cities. Intercity flight and train information were obtained from the official platforms of the Railway Customer Service Center of China and the Civil Aviation Administration of China. The traffic eigenvector centrality indicates the centrality of a vertex (i.e., city) in the transportation network based on the weighted sum of the centralities of its neighbors (for more details, see Additional file 1: Text S3) [27]. A high traffic eigenvector centrality indicates that a city has well-developed transportation links with other cities.

### Statistical analysis

Initially, the present state and geographical distribution of the nephrology workforce in mainland China were described, including the number of nephrologists and the proportion of nephrologists relative to all physicians.

Subsequently, utilizing real-world patient mobility data from the CKD population, we evaluated the optimal capacity of the nephrology workforce in China. Multivariate Poisson regression models were employed to analyze the association between the number of patient mobility instances and the nephrology workforce in source or destination cities. The dependent variable in our study, namely the number of cross-city hospitalizations, adheres to the four assumptions of a Poisson regression model: the dependent variable is the count of events, all events are independent, the average occurrence rate of events is independent of any event, and two events cannot occur simultaneously. The regression models were adjusted for the following covariates, healthcare capacity, economic status, and transportation infrastructure in both source and destination cities, health insurance in the source city, and the shortest highway distance between source and destination cities. Results from univariate Poisson regression models were also provided for model comparison. To reduce the patient mobility for CKD and meet the healthcare needs of patients with CKD in a city simultaneously, we postulated that a city's optimal nephrology workforce capacity corresponds to the point where the positive effect of the workforce on patient inflow is relatively minimal, and the negative effect on patient outflow is pronounced. Consequently, we investigated the non-linear relationships between the nephrology workforce and patient mobility for CKD to evaluate the optimal workforce capacity. Natural spline functions of the nephrology workforce (with three degrees of freedom) were used to estimate these non-linear effects.

As part of the sensitivity analyses, we also explored the non-linear effects of the nephrology workforce on CKD patient mobility, stratified by cross-province and within-province mobility. Additionally, we conducted analyses using data spanning a 2-year period from 2017 to 2018 to assess the robustness of the results over different study periods.

In this study, the estimated effects of the nephrology workforce on patient mobility for CKD are presented as incidence rate ratios (IRRs) with 95% confidence intervals (CIs). The IRR represents the ratio of the incidence rate of patient mobility for CKD in the exposed group compared to that in the control group. All *P*-values are two-sided, and a *P*-value < 0.05 was deemed statistically significant. Analyses were conducted using R software (version 4.1.3; R Development Core Team, Vienna, Austria), and the models were developed employing the *mgcv* (version 1.8–39) and *splines* (version 4.1.3) packages.

## Results

### Status of nephrology workforce in China

**Fig. 1:**
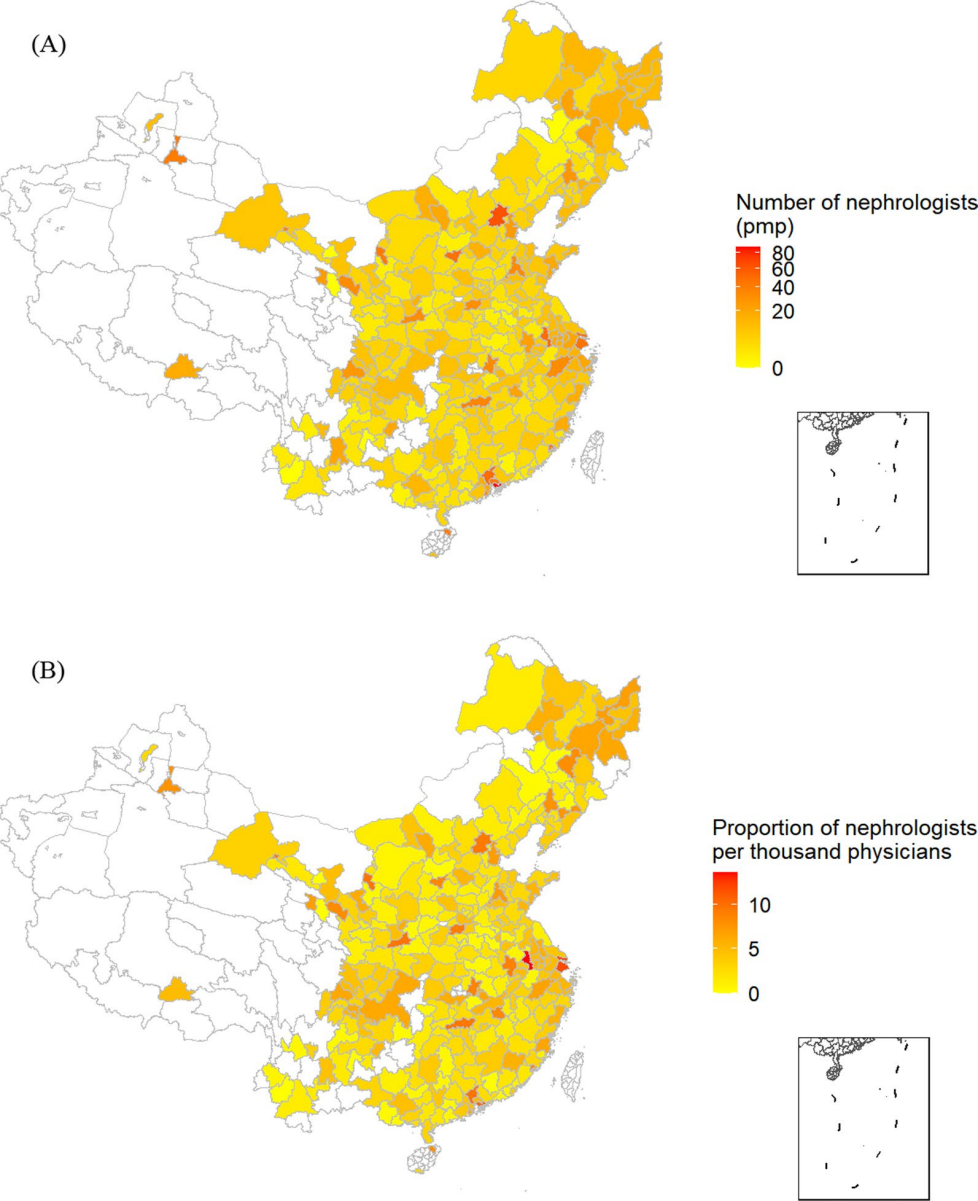
Spatial distribution of density of nephrology workforce in China. In **A** the white areas in the map indicate that the numbers of population in these cities were not available, and thus numbers of nephrologists per million population cannot be calculated. In **B** the white areas in the map indicate that the numbers of physicians in these cities were not available, and thus proportions of nephrologists per thousand physicians cannot be calculated

Figure 1 illustrates the geographical distribution of the nephrology workforce in mainland China. A total of 12 894 nephrologists were identified, 4258 (33.0%) of whom are located in East China. Consequently, there were 9.13 nephrologists per million population (pmp) in practice, with considerable city-level variation ranging from 0.16 to 88.79 (Fig. 1A). Considering the prevalence of CKD in China of 10.8%, the ratio of nephrologists to the estimated CKD population was 84.57 pmp. The proportion of nephrologists among all physicians varied from 0.18 to 13.6 per thousand (Fig. 1B).

### Characteristics of patient mobility for CKD

Of the 6 415 559 hospitalizations for patients with CKD, 21.3% were categorized as cross-city hospitalizations. Over a 5-year period from 2014 to 2018, 7441 city-level paths of patient mobility with more than five hospitalizations each were identified (Table 1). Among these paths, a total of 1 332 189 cross-city hospitalization records for CKD were included in the Poisson regression analyses, and 1 027 238 (77.1%) of them were within the same province. Destination cities, when compared to source cities, had a higher average number of nephrologists (22.8 vs. 14.1 pmp) and a higher proportion of nephrologists among all physicians (6.0 vs. 4.2 per thousand).

**Table 1:** Basic characteristics of patient mobility for CKD. Detailed definitions of variables were provided in Additional file 1: Table S2

	All	Within-province	Cross-province	Missing^†^	P^*^
Number of city-level paths of mobility	7441	1990 (26.7%)	5451 (73.3%)		
Number of cross-city hospitalizations (total)	1 332 189	1 027 238 (77.1%)	304 951 (22.9%)		
Number of cross-city hospitalizations (median [interquartile range])	16.0 [9.0–44.0]	30.0 [12.0–172.75]	13.0 [8.0–30.0]		
Source city					
Number of nephrologists (pmp)	14.1 ± 16.8	9.4 ± 11.5	15.7 ± 18.1	686	0.026
Proportion of nephrologists per thousand physicians	4.2 ± 2.9	3.4 ± 2.4	4.5 ± 3.0	686	0.101
UBMI coverage proportion (%)	37.2 ± 51.9	26.9 ± 34.8	40.8 ± 56.1	686	0.216
Number of hospital beds (pmp)	5231.5 ± 2077.5	4755.9 ± 1760.4	5396.7 ± 2152.5	686	0.005
Number of physicians (pmp)	2690.0 ± 1517.9	2302.6 ± 1123.3	2824.6 ± 1611.2	686	0.007
Traffic eigenvector centrality	2.1 ± 2.4	1.7 ± 2.1	2.3 ± 2.5	59	0.924
Average annual population (million)	5.9 ± 4.6	4.9 ± 2.6	6.2 ± 5.1	686	0.292
GDP per capita (¥1000)	61.9 ± 42.6	53.9 ± 36.2	64.7 ± 44.3	686	0.302
Destination city					
Number of nephrologists (pmp)	22.8 ± 19.2	12.4 ± 13.8	26.4 ± 19.5	307	< 0.001
Proportion of nephrologists per thousand physicians	6.0 ± 3.2	4.1 ± 2.5	6.7 ± 3.1	307	< 0.001
Number of hospital beds (pmp)	6220.6 ± 2190.0	5218.7 ± 1986.9	6563.7 ± 2150.8	307	< 0.001
Number of physicians (pmp)	3275.0 ± 1614.4	2561.0 ± 1333.9	3519.5 ± 1629.5	307	< 0.001
Traffic eigenvector centrality	2.9 ± 2.8	1.7 ± 2.2	3.3 ± 2.8	22	< 0.001
Average annual population (million)	7.3 ± 5.1	5.1 ± 2.6	8.1 ± 5.5	307	< 0.001
GDP per capita (¥1000)	76.6 ± 42.8	58.7 ± 38.1	82.7 ± 42.7	307	< 0.001
Between cities					
Highway distance (km)	998.7 ± 816.9	315.1 ± 243.0	1249.5 ± 810.0	577	< 0.001

^†^The number of missing values out of the 7441 instances, namely, city-level paths

*P values of Spearman’s Rank Correlation Test with the number of cross-city hospitalizations

### Impact of nephrology workforce on patient mobility

Table 2 presents the results of both univariate and multivariate Poisson regression analyses examining the impact of the nephrology workforce on CKD patient mobility. After adjusting for healthcare capacity, health insurance, economic status, and transportation infrastructure, the Poisson regression models indicated that the number of nephrologists in both the source city (IRR 0.99, per 1 pmp increase) and the destination city (IRR 1.07, per 1 pmp increase) were independently associated with patient mobility. Likewise, the proportion of nephrologists per thousand physicians in both the source city (IRR 0.90) and the destination city (IRR 1.35) were independently associated with patient mobility.

**Table 2:** Effect of nephrology workforce on patient mobility for CKD

Model	Number of nephrologists				Proportion of nephrologists per thousand physicians			
	Source city		Destination city		Source city		Destination city	
	IRR	*P*	IRR	*P*	IRR	*P*	IRR	*P*
Unadjusted	0.97 [0.97, 0.97]	< 0.001	1.02 [1.02, 1.02]	< 0.001	0.81 [0.81, 0.81]	< 0.001	1.16 [1.16, 1.16]	< 0.001
Model 1	0.97 [0.97, 0.97]	< 0.001	1.02 [1.02, 1.02]	< 0.001	0.85 [0.85, 0.85]	< 0.001	1.17 [1.17, 1.17]	< 0.001
Model 2	0.97 [0.97, 0.97]	< 0.001	1.04 [1.04, 1.04]	< 0.001	0.82 [0.82, 0.82]	< 0.001	1.18 [1.18, 1.18]	< 0.001
Model 3	0.99 [0.99, 0.99]	< 0.001	1.07 [1.07, 1.07]	< 0.001	0.90 [0.90, 0.90]	< 0.001	1.35 [1.35, 1.35]	< 0.001

Model 1: adjusted for characteristics of source city

Model 2: adjusted for characteristics of destination city

Model 3: adjusted for characteristics of source city, destination city, and between cities

**Fig. 2:**
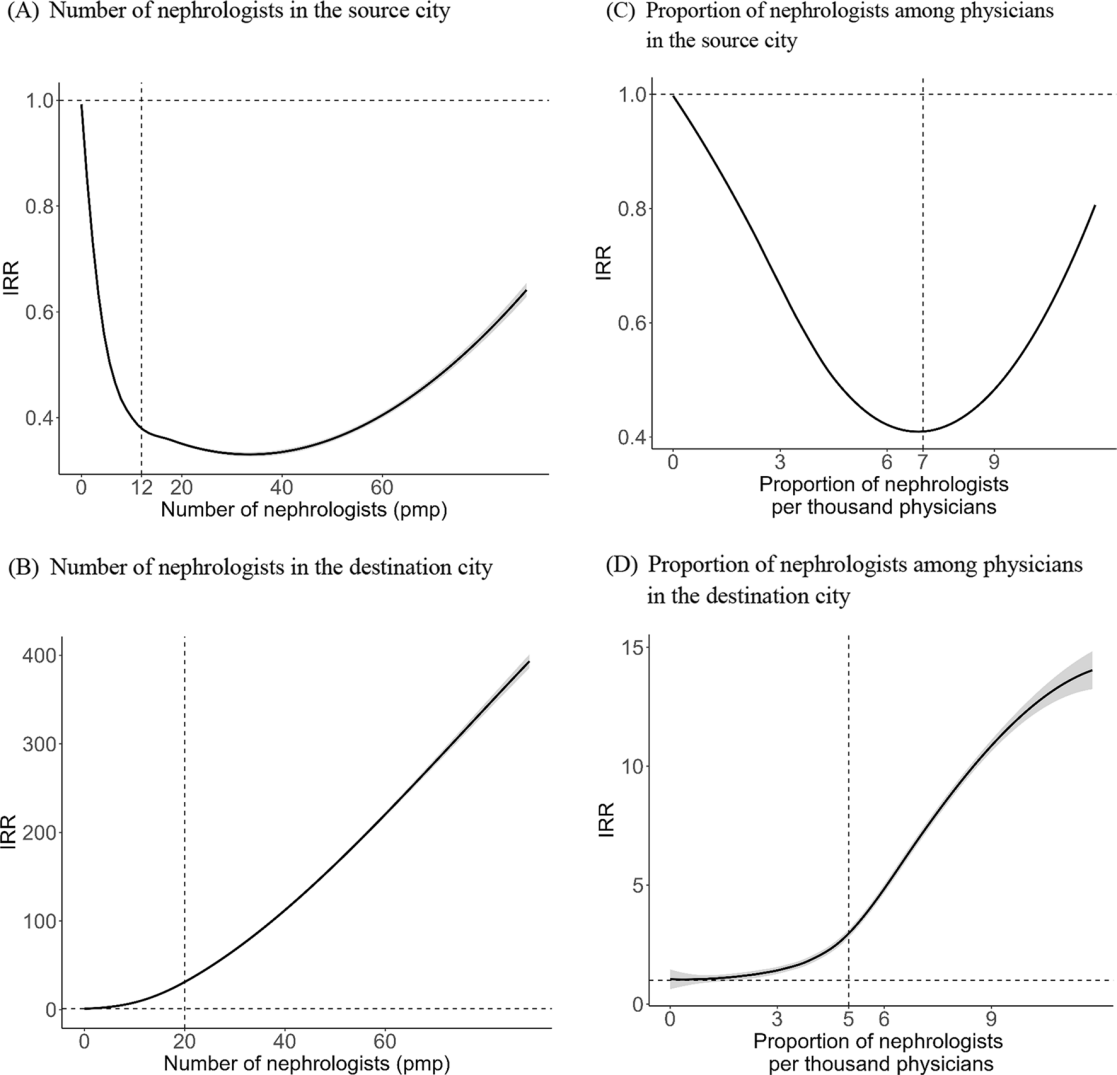
Non-linear effect of nephrology workforce on patient mobility for CKD. **A** Number of nephrologists in the source city. **B** Number of nephrologists in the destination city. **C** Proportion of nephrologists among physicians in the source city. **D** Proportion of nephrologists among physicians in the destination city

Figure 2 reveals the non-linear effects of the nephrology workforce on patient mobility for CKD. A relatively stable IRR was observed when the number of nephrologists exceeded 12 pmp in the source city (Fig. 2A), whereas a rapidly increasing IRR was observed when the number of nephrologists exceeded 20 pmp in the destination city (Fig. 2B). Similarly, a relatively stable IRR was observed when the proportion of nephrologists among physicians exceeded 7 per thousand in the source city (Fig. 2C), and a sharply increasing IRR was observed when this proportion exceeded 5 per thousand in the destination city (Fig. 2D).

When stratified by cross-province and within-province mobility (Additional file 1: Fig. S1), the nephrology workforce in the source city had a more substantial impact on cross-province mobility compared to within-province mobility for CKD. Additionally, a threshold effect was discerned in the non-linear relationship between the nephrology workforce in the destination city and cross-province patient mobility.

### Sensitivity analyses

The sensitivity analysis demonstrated that the results were robust with respect to the study period (Additional file 1: Fig. S2). The non-linear effects of the nephrology workforce on patient mobility for CKD, based on data over a 2-year period (2017–2018), displayed a similar pattern to those based on data obtained over a 5-year period (2014–2018).

## Discussion

This study is pioneering in quantitatively describing the current status and distribution of the nephrology workforce in China, as well as evaluating its optimal capacity using real-world patient mobility data. There were approximately 9.13 nephrologists pmp in practice, with significant geographical variations. The ratio of nephrologists to the estimated CKD population was 84.57 pmp. Based on the estimated non-linear effects of the nephrology workforce on patient mobility for CKD, 12–20 nephrologists pmp might be optimal for addressing local healthcare needs in China. This study offers data-driven evidence that can inform policy-making to establish an equitable nephrology workforce capacity capable of effectively tackling the growing burden of CKD in China.

Several studies have explored the state of the nephrology workforce worldwide through questionnaires, emphasizing the gaps and shortcomings in workforce availability and quality [5, 6, 21, 28, 29]. However, there is a dearth of evidence based on real-world data regarding the exact number of nephrology workforce, especially in China, where a central registration system for nephrologists is absent. A joint questionnaire investigation conducted by ASN, ERA-EDTA, and ISN reported a global median of 9.1 nephrologists pmp (95% CI 2.3–24.9), with the median number of nephrologists per million CKD population ranging from 8 in sub-Saharan Africa to 231 in North America [6]. It was also reported that in China, the number of nephrologists, nephrologist density, and the ratio of nephrologists to the CKD population were 8500, 6.2 pmp, and 64 pmp, respectively [6]. Utilizing real-world online data, our study is the first to report the exact number (*n* = 12 894) of practicing nephrologists in China, and estimated the nephrologist density at 9.13 pmp, and the ratio of nephrologists to CKD population at 84.57 pmp. Our results align with previous studies. While nephrologist density in China has reached the global median, the ratio of nephrologists to the CKD population is considerably lower than that in high-income countries, which is a concern given China’s large CKD population. Expanding the nephrology workforce is critical for addressing the escalating burden of kidney diseases in China.

Alongside the identified shortage of the nephrologists in China, there is a geographical disparity in their distribution. The data suggest that in many regions of the country, individuals requiring kidney care may receive either subpar care or no care at all. A supply side-oriented resource allocation might account for this geographical disparity in the nephrology workforce, resulting in a concentration of limited healthcare resources in more developed areas [30]. Given the significant financial and time investment needed to train qualified nephrologists, rapidly scaling up the existing nephrology workforce might not be feasible, particularly in underdeveloped areas. Distributing the nephrology workforce more evenly could be a viable solution to this issue. Several strategies could be considered to enhance the availability and equity of the nephrology workforce, such as incentivizing nephrologists to practice in underdeveloped areas by offering professional development opportunities, bolstering allied health services in nephrology, and expanding the role of primary care physicians in kidney care management [21, 31–33].

Assessing workforce adequacy and identifying the optimal capacity are crucial for efficient and equitable allocation of the nephrology workforce. However, there is no standardized framework to assess workforce adequacy, and limited literature has focused on nephrology workforce size recommendations due to data constraints [6, 34]. Specifically, for accurate evaluation of the optimal capacity of a nephrology workforce for a given region, an understanding of the heterogeneity in healthcare needs and the establishment of minimum baselines for nephrologists to guide workforce development are needed. The conventional method for healthcare workforce evaluation relies on national workforce standards for clinical specialists and national population projections, which do not account for demand heterogeneity in clinical practice and can be somewhat subjective [35]. Our study introduces a novel method for evaluating nephrology workforce capacity, specifically, employing real-world cross-city mobility data of patients with CKD as an evaluation metric. Through this method, we estimated that having 12–20 nephrologists pmp could be optimal for addressing local healthcare needs in China. Patient mobility has been used as a proxy measure to evaluate the availability and quality of hospital services and has been widely studied in the context of healthcare policy-making. For example, Koylu et al. [8] performed a systematic analysis of patient mobility at the provincial level in Turkey based on a national healthcare system, to identify functional medical regions and mismatches between planned regional service delivery and observed service utilization. Pecoraro et al. [10] used patient mobility data to obtain insight into the distribution of hospital accessibility in Italy, guiding efforts to enhance equity in healthcare service provision nationwide. Moreover, healthcare capacity has been identified as a significant factor influencing patient mobility in health service studies [9, 24]. As such, we believe our estimation of optimal nephrology workforce capacity based on patient mobility holds practical value for nephrology workforce development in China.

It is important to acknowledge certain limitations of our study. First, the nephrology workforce in practice might have been underestimated in this study, since data from online healthcare platforms may not encompass nephrologists in regions with underdeveloped healthcare digitalization. Second, only mobility data for patients in tertiary hospitals and city-level paths with more than five hospitalizations during the study period were analyzed, which could have introduced selection biases in our estimates. Nevertheless, patients seeking cross-city healthcare services often pursue high-quality services at tertiary hospitals, and hospitalization inherently reflects the disease burden of CKD. Therefore, the representativeness of our data is likely sufficient. Third, characterization of the nephrology workforce was based solely on the number of nephrologists, while aspects such as the quality, efficiency, or accessibility of the nephrology workforce were not considered due to data limitations. Moreover, the reliability of the proposed method for evaluating nephrology workforce capacity, specifically, employing real-world cross-city mobility data of patients with CKD as an evaluation metric, needs further validation in future studies. Lastly, we were unable to control for confounding factors such as individual's ability or motivation to seek cross-city healthcare services, or cultural differences in the regression models; additional individual-level analyses are warranted. Besides, due to data limitations, the NRCMS coverage proportion was not factored into the assessment of the confounding effects of a city's health insurance capacity.

## Conclusions

In conclusion, this study found significant geographic variations in the nephrology workforce across China, and having 12–20 nephrologists pmp might be optimal for addressing local healthcare needs. Our study offers real-world evidence that can assist policymakers in optimizing the development and allocation of the nephrology workforce. Given the escalating burden of kidney disease in China, there is a pressing need to expand the current nephrology workforce. Moreover, employing a data-driven approach is essential for ensuring a balance between efficiency and equity in the distribution and utilization of the nephrology workforce.

### Supplementary Information

**Additional file 1:** **Text S1.** Methods of obtaining nephrology workforce data. **Text S2. **Validation of nephrology workforce data. **Text S3.** Traffic eigenvector centrality. **Table S1.** The International Classification of Diseases-10 coding of CKD. **Table S2.** Definitions of variables. **Figure S1.** Non-linear effect of nephrology workforce on patient mobility for CKD stratified by cross-province and within-province mobility. **A** Number of nephrologists in the source city on cross-province mobility. **B** Number of nephrologists in the source city on within-province mobility. **C** Number of nephrologists in the destination city on cross-province mobility. **D** Number of nephrologists in the destination city on within-province mobility. **Figure S2.** Non-linear effect of nephrology workforce on patient mobility for CKD in sensitivity analysis. **A** Number of nephrologists in the source city. **B** Number of nephrologists in the destination city. **C** Proportion of nephrologists among physicians in the source city. **D** Proportion of nephrologists among physicians in the destination city.

## Data Availability

Data may be obtained from a third party and are not publicly available. The data that support the findings of this study are available from the Bureau of Medical Administration and Medical Service Supervision, National Health Commission of China, but restrictions apply to the availability of these data, which were used under license for the current study and so are not publicly available. Data are however available from the authors upon reasonable request and with permission of the Bureau of Medical Administration and Medical Service Supervision, National Health Commission of China.

## Abbreviations

CKD: Chronic kidney disease
pmp: Per million population
IRR: Incidence risk ratio
ASN: American Society of Nephrology
ERA-EDTA: European Renal Association–European Dialysis and Transplant Association
ISN: International Society of Nephrology
HQMS: Hospital Quality Monitoring System
ICD-10: International Classification of Diseases-10
UBMI: Urban Basic Medical Insurance
GDP: Gross domestic product
