# Supplementary material for: Nephrology workforce in China: describing current status and evaluating the optimal capacity based on real-world data
Source: Hum Resour Health. 2023 Aug 8;21:62. doi: 10.1186/s12960-023-00851-3 (PMC10410773; doi:10.1186/s12960-023-00851-3)
Supplement: Supplementary file 1 — Additional file 1: Text S1. Methods of obtaining nephrology workforce data. Text S2. Validation of nephrology workforce data. Text S3. Traffic eigenvector centrality. Table S1. The International Classification of Diseases-10 coding of CKD. Table S2. Definitions of variables. Figure S1. Non-linear effect of nephrology workforce on patient mobility for CKD stratified by cross-province and within-province mobility. A Number of nephrologists in the source city on cross-province mobility. B Number of nephrologists in the source city on within-province mobility. C Number of nephrologists in the destination city on cross-province mobility. D Number of nephrologists in the destination city on within-province mobility. Figure S2. Non-linear effect of nephrology workforce on patient mobility for CKD in sensitivity analysis. A Number of nephrologists in the source city. B Number of nephrologists in the destination city. C Proportion of nephrologists among physicians in the source city. D Proportion of nephrologists among physicians in the destination city. [file 12960_2023_851_MOESM1_ESM.docx]

# **Supplementary Material**

**Text S1. Methods of obtaining nephrology workforce data**

Using web crawlers and natural language processing techniques, we obtained data regarding nephrology workforce in China from two leading online healthcare platforms, Haodaifu (“Good Doctor” in English, www.haodf.com) and WeDoctor ([www.guahao.com](http://www.guahao.com)). The brief process is as follows. First, we collected extensive information of all physicians from the two online healthcare platforms using web crawlers, including the locations, seniority, medical specialty, affiliated hospital, and affiliated department. Second, we identified nephrologists from all physicians based on natural language processing techniques. A physician was identified as a nephrologist if the name of his/her affiliated department contains nephrology-related keywords (i.e., “肾” or “透析” or “血液净化” or “血透” or “腹透” or “血浆净化” in Chinese). We conducted a manually double-check based on the medical specialty descriptions of physicians, and excluded those misclassified physicians. Third, we standardized the names of the nephrologists’ affiliated hospitals, and excluded the duplicated records of nephrologists using their names and medical specialties. Based on the standardized data of all nephrologists, we calculated number of nephrologists and proportion of nephrologists among physicians for each city.

**Text S2. Validation of nephrology workforce data**

We performed manual data validation using information from 10 urban hospitals in four developed cities (Beijing, Shanghai, Guangzhou, and Shenzhen) and 5 hospitals in five rural regions, and checked the agreement between the number of nephrologists per hospital identified using web crawlers and natural language processing techniques and that manually extracted from the hospital official websites. The agreement of the number of nephrologists was measured using the mean absolute error (MAE), which was calculated as follows:

$$\mathrm{MAE}=\frac{\sum\left| N_{i}-N_{\mathrm{manually},i} \right|}{n}$$

where *N_i_* denotes the number of nephrologists in the *i*th hospital identified using web crawlers and natural language processing techniques from online healthcare platforms, *N*_manually_*_,i_* denotes the number of nephrologists in the *i*th hospital manually extracted from the hospital official website, and *n* denotes the number of hospitals for data validation.

**Text S3. Traffic eigenvector centrality**

Eigenvector centrality was firstly used in graph theory as a measure of the influence a vertex has on a network. More recently, researchers across many fields have analyzed the applications and extensions of eigenvector centrality in a variety of domains, and traffic eigenvector centrality has been a commonly-used measure of traffic development in recent studies.(Azimifar, Soltani sarvestani et al. 2020, Reza, Ferreira et al. 2022) The traffic eigenvector centrality measures the centrality of a vertex (i.e., city) in the traffic network based on the weighted sum of centralities of its neighbors. For a vertex *v*, the traffic eigenvector centrality *x_v_* could be calculated using a power iteration strategy as follows.

$$x_{v}=\frac{1}{\lambda}\sum_{t\in M(v)} x_{t}=\frac{1}{\lambda}\sum_{t\in V} a_{v,t}x_{t}$$

For a given traffic network *G* = (*V*, *E*), where *V* denotes the vertices and *E* denotes the edges, let *A* = (*a_v_*_,_*_t_*) be the adjacency matrix, i.e., *a_v_*_,_*_t_* = 1 if vertex *v* is linked to vertex *t*, and *a_v_*_,_*_t_* = 0 otherwise. *M*(*v*) denotes the set of neighbors of *v* and *λ* is a constant.

**References**

Azimifar, Z., M. Soltani sarvestani, A. A. Safavi and G. Habibagahi (2020). "Expanding the eigenvector centrality for multi-layer graphs and its application in managing traffic and urban infrastructure." Quarterly Journal of Transportation Engineering: -.

Reza, S., M. C. Ferreira, J. J. M. Machado and J. M. R. S. Tavares (2022). "Road networks structure analysis: A preliminary network science-based approach." Annals of Mathematics and Artificial Intelligence.

Table S1. The International Classification of Diseases-10 coding of CKD.

| Etiology of CKD | All editions | China edition | Beijing edition | Clinic edition |
| --- | --- | --- | --- | --- |
| 1. Diabetes mellitus |  |  |  |  |
| Type 1 diabetes mellitus with renal complications | E10.2+ N08.3 |  |  |  |
| Type 2 diabetes mellitus with renal complications | E11.2+ N08.3 |  |  |  |
| Unspecified diabetes mellitus with renal complications | E14.2 |  |  |  |
| Malnutrition-related diabetes mellitus with renal complications |  | E12.200+N08.3 |  | E12.200 |
| Other specified diabetes mellitus with renal complications |  | E13.2 |  | E13.200 |
| 2. Hypertensive diseases |  |  |  |  |
| Hypertensive renal disease with renal failure | I12 |  |  |  |
| Hypertensive heart and renal disease with (congestive) heart failure | I13 |  |  |  |
| Pregnancy with hypertensive heart and renal disease | O10.301 |  |  |  |
| Pregnancy with essential hypertension and proteinuria | O11.x01 |  |  |  |
| Pre-existing hypertensive renal disease during pregnancy,  childbirth and puerperium |  | O10.200 |  | O10.200 |
| Pregnancy with hypertensive renal disease |  | O10.201 |  | O10.201 |
| Pre-existing hypertensive heart and renal disease during pregnancy,  childbirth and puerperium |  | O10.300 |  | O10.300 |
| Pre-existing hypertension with proteinuria |  | O11.x00 |  | O11.x00 |
| 3. Glomerular diseases |  |  |  |  |
| Recurrent and persistent hematuria | N02 |  |  |  |
| Chronic nephritic syndrome | N03 |  |  |  |
| Nephrotic syndrome | N04 |  |  |  |
| Unspecified nephritic syndrome | N05 |  |  |  |
| Isolated proteinuria with specified  morphological lesion | N06 |  |  |  |
| Persistent proteinuria, unspecified | N39.1 |  |  |  |
| 4. Renal tubulointerstitial diseases |  |  |  |  |
| Chronic tubulointerstitial nephritis | N11 |  |  |  |
| Tubulointerstitial nephritis, not specified as acute or chronic | N12 |  |  |  |
| Drug- and heavy-metal-induced tubulointerstitial and tubular conditions | N14 |  |  |  |
| Renal tubulointerstitial disorders in diseases classified elsewhere | N16 |  |  |  |
| Other specified disorders of carbohydrate metabolism | E74.8 |  |  |  |
| Disorders of amino-acid transport | E72.0 |  |  |  |
| Nephrogenic diabetes insipidus |  | N25.1 |  | N25.1 |
| Renal tubule acidosis | N25.8 |  |  |  |
| Balkan nephropathy |  | N15.000 | N15.001 | N15.000 |
| Renal tubulointerstitial disease, specified |  | N15.800 |  | N15.800 |
| Renal granuloma |  | N15.801 |  | N15.801 |
| Renal tubulointerstitial disease |  | N15.900 |  | N15.900 |
| Impaired renal tubular function-related disease |  | N25.9 |  | N25.9 |
| Liddle syndrome |  | I15.101 |  | I15.101 |
| Urate nephropathy |  | M10.001+N16.8 | N28.905 | M10.001+N16.8 |
| Systemic lupus erythematosus + renal tubulointerstitial diseases |  | M32.102+N16.4 | M32.113+N16.4 | M32.102+N16.4 |
| Sicca syndrome + renal tubulointerstitial diseases |  | M35.006+N16.4 | M35.005+N16.4 | M35.006+N16.4 |
| 5. Obstructive nephropathy |  |  |  |  |
| Hydronephrosis with ureteropelvic junction obstruction | N13.0 |  |  |  |
| Hydronephrosis with ureteral stricture, not elsewhere classified | N13.1 |  |  |  |
| Hydronephrosis with renal and ureteral calculous obstruction |  | N13.2 | N13.2 | N13.200 |
| Other obstructive nephropathy |  | N13.8 | N13.8 | N13.801 |
| 6. Other related diagnosis |  |  |  |  |
| Hereditary nephropathy, not elsewhere classified |  | N07 | N07.901 | N07 |
| Glomerular disorders in diseases classified elsewhere | N08, exclude N08.5 |  |  |  |
| Renal agenesis and other reduction defects of kidney | Q60 |  |  |  |
| Polycystic kidney, autosomal recessive | Q61.1 |  |  |  |
| Polycystic kidney, autosomal dominant | Q61.2 |  |  |  |
| Polycystic kidney, unspecified | Q61.3 |  |  |  |
| Medullary cystic kidney, sponge kidney NOS | Q61.5 |  |  |  |
| Lobulated, fused and horseshoe kidney | Q63.1 |  |  |  |
| Congenital malformation of kidney, unspecified | Q63.9 |  |  |  |
| Gout due to impairment of renal function |  | M10.300 | M10.393 | M10.300 |
| Unspecified contracted kidney | N26 |  |  |  |
| Ischemia and infarction of kidney | N28.0 |  |  |  |
| Other specified disorders of kidney and ureter | N28.8 |  |  |  |
| Disorders of kidney and ureter, unspecified | N28.9 |  |  |  |
| Congenital renal failure |  | P96.0 | P96.0 | P96.000 |
| Extrarenal uremia | R39.2 |  |  |  |
| Aortic arch syndrome + renovascular hypertension |  | M31.4 + I15.0 | I77.604 + I15.0 | I77.600x004 + I15.0 |
| Goodpasture syndrome | M31.001 |  |  |  |
| Renal osteodystrophy | N25.0 |  |  |  |
| Failure and rejection of renal transplantation | T86.1 |  |  |  |
| Hemolytic uremic syndrome | D59.3 |  |  |  |
| Dialysis | Z49 |  |  |  |
| Renal allergic purpura | D69.005+N08.2 |  |  |  |
| Lupus nephritis |  | M32.101+N08.5 | M32.105+N08.5 | M32.101+N08.5 |
| Goodpasture syndrome-related glomerulonephritis |  | M31.003+N08.5 |  | M31.003+N08.5 |
| Antiglomerular basement membrane antibody-related disease |  | M31.002+N08.5 | M31.005+N08.5 | M31.002+N08.5 |
| Microscopic polyangitis |  | M31.700 | M31.701 | M31.700 |
| ANCA-related nephritis |  | M31.701+N08.5 | M31.802 | M31.701+N08.5 |
| Thrombotic thrombocytopenic purpura-related glomerulonephritis |  | M31.102+N08.5 |  | M31.102+N08.5 |
| Wegener’s granulomatosis-related glomerulonephritis |  | M31.303+N08.5 |  | M31.303+N08.5 |
| Pregnancy with nephrotic syndrome |  | O26.801 | O26.811 | O26.801 |
| Pregnancy with glomerulonephritis |  | O26.804 | O26.812 | O26.804 |
| Pregnancy with renal failure |  | O26.802 | O26.813 | O26.802 |
| HBV-related nephritis |  | B18.103+N08.0 | B18.102 | B18.103+N08.0 |
| HCV-related nephritis |  | B18.205+N08.0 | B18.208 | B18.205+N08.0 |
| Cryoglobulinaemia-related glomerulonephritis |  | D89.101+N08.2 |  | D89.101+N08.2 |
| Hereditary amyloidosis nephropathy |  | E85.002 | E85.003 | E85.002 |
| Amyloidosis-related nephropathy |  | E85.411+N29.8 | E85.410+N08.4 | E85.411+N29.8 |
| Psoriatic nephritis |  | L40.803+ | L40.802+N05.9 | L40.800x002+N05.9 |
| Kidney injury-related gout |  | M10.300 | M10.393 | M10.300 |
| Syphilitic nephritis |  |  | A52.712+N08.0 | A52.700x012+N08.0 |
| Lupus kidney injury |  |  | M32.112+N08.5 |  |
| Lupus nephritis |  | M32.101+N08.5 | M32.105+N08.5 | M32.101+N08.5 |
| Lupus tubulointerstitial kidney |  | M32.102+N16.4 | M32.113+N16.4 | M32.102+N16.4 |
| Gouty nephropathy |  |  | M10.391 | M10.300x091 |
| Gouty nephrolithiasis |  | M10.005+N22.8 | M10.392 | M10.005+N22.8 |

Table S2. Definitions of variables.

| Variables | Definitions |
| --- | --- |
| City-level path of mobility | A unique path of patient mobility from a source city to a destination city. |
| Number of nephrologists (per million population [pmp]) | The ratio of the number of nephrologists in a city to the average annual population in a city. |
| Proportion of nephrologists per thousand physicians | The ratio of the number of nephrologists in a city to the number of all physicians in a city. |
| UBMI coverage proportion (%) | The ratio of the number of population insured by UBMI to the average annual population in a city. |
| Number of hospital beds (pmp) | The ratio of the number of hospital beds in a city to the average annual population in a city. |
| Number of physicians (pmp) | The ratio of the number of all physicians in a city to the average annual population in a city. |
| Traffic eigenvector centrality | The centrality of a vertex (i.e., city) in the traffic network calculated as the weighted sum of centralities of its neighbors (for more details, see Supplementary Text S3). |
| Average annual population (million) | The mean of the number of annual population during the study period (2014-2018). |
| GDP per capita (¥1,000) | The mean of annual GDP per capita during the study period (2014-2018). |

1. Number of nephrologists in the source city on cross-province mobility


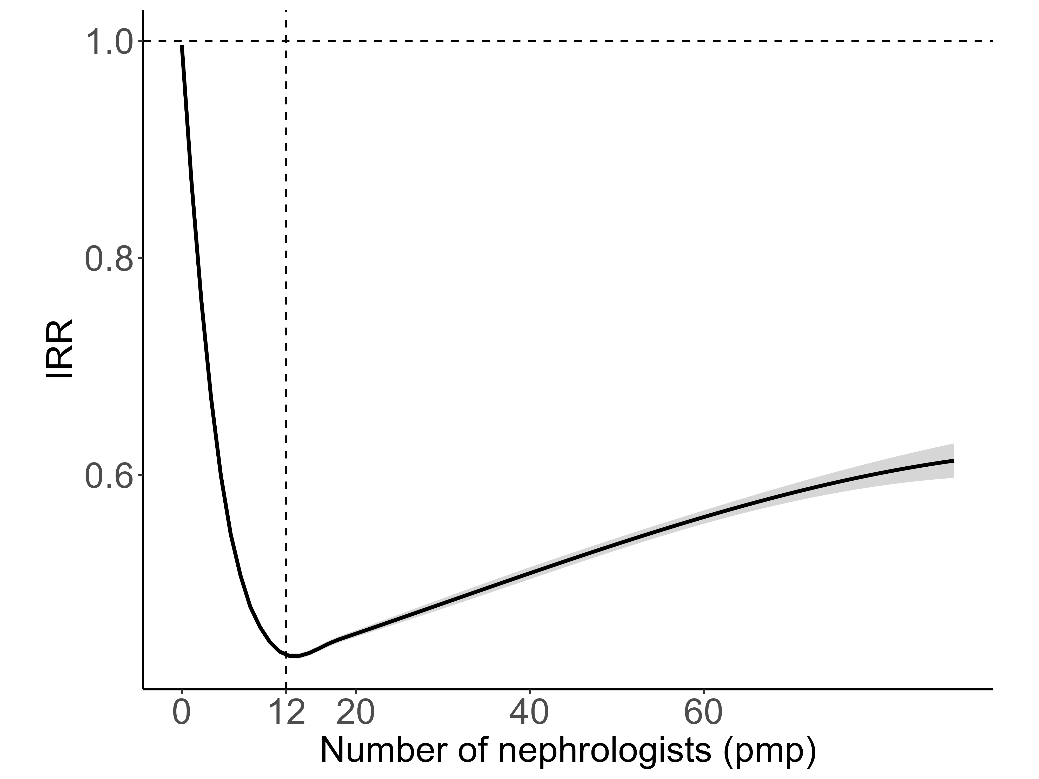


1. Number of nephrologists in the source city on within-province mobility


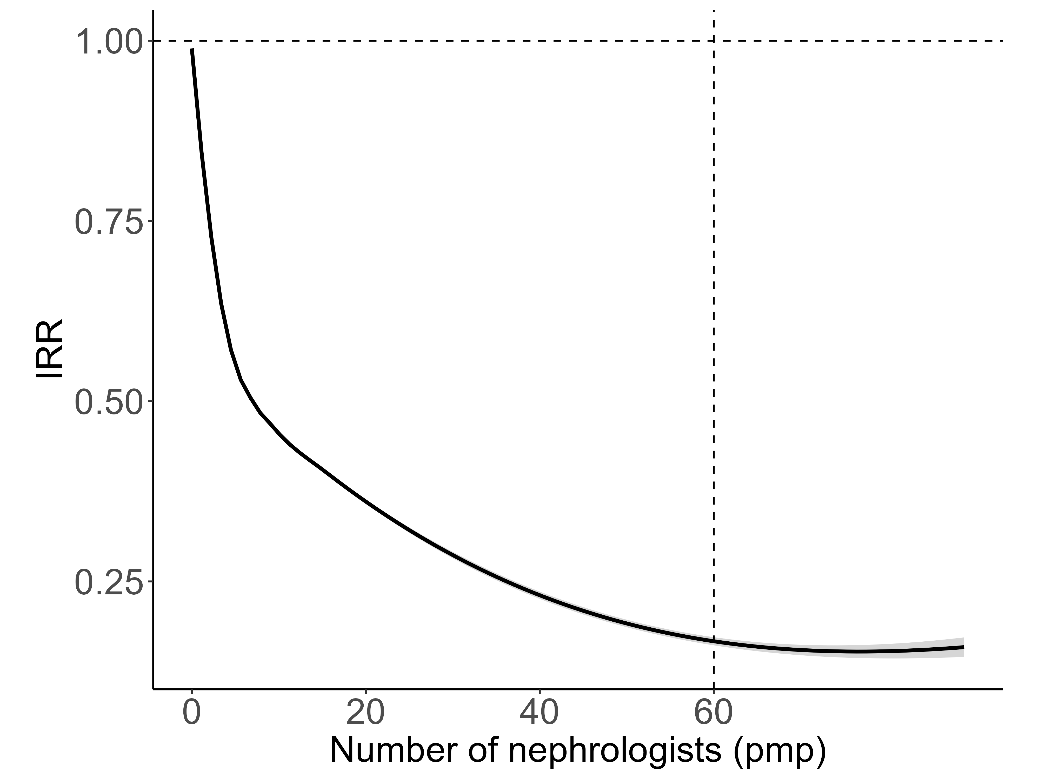


1. Number of nephrologists in the destination city on cross-province mobility


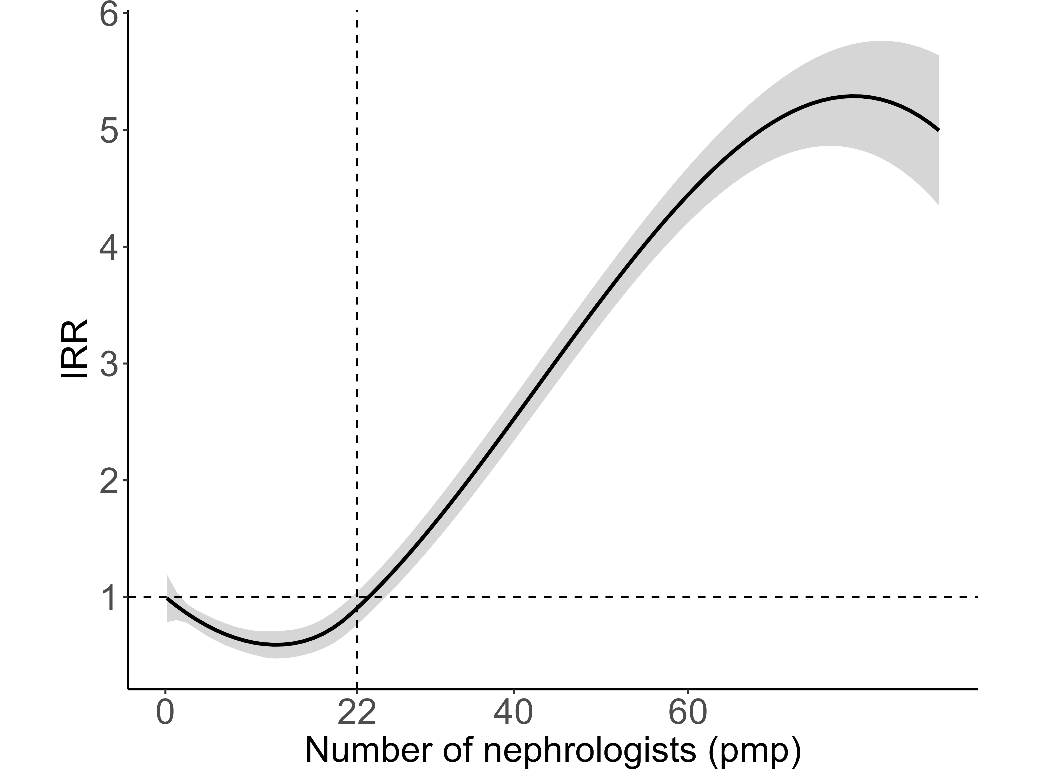


1. Number of nephrologists in the destination city on within-province mobility


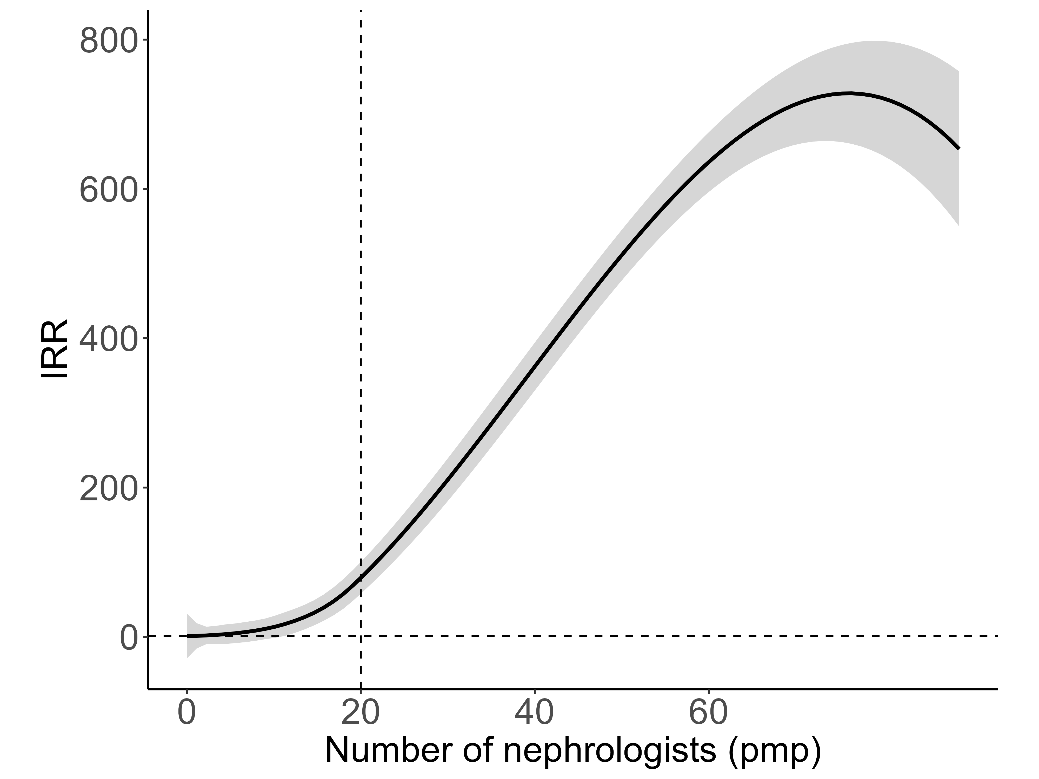


Figure S1. Non-linear effect of nephrology workforce on patient mobility for CKD stratified by cross-province and within-province mobility.

1. Number of nephrologists in the source city


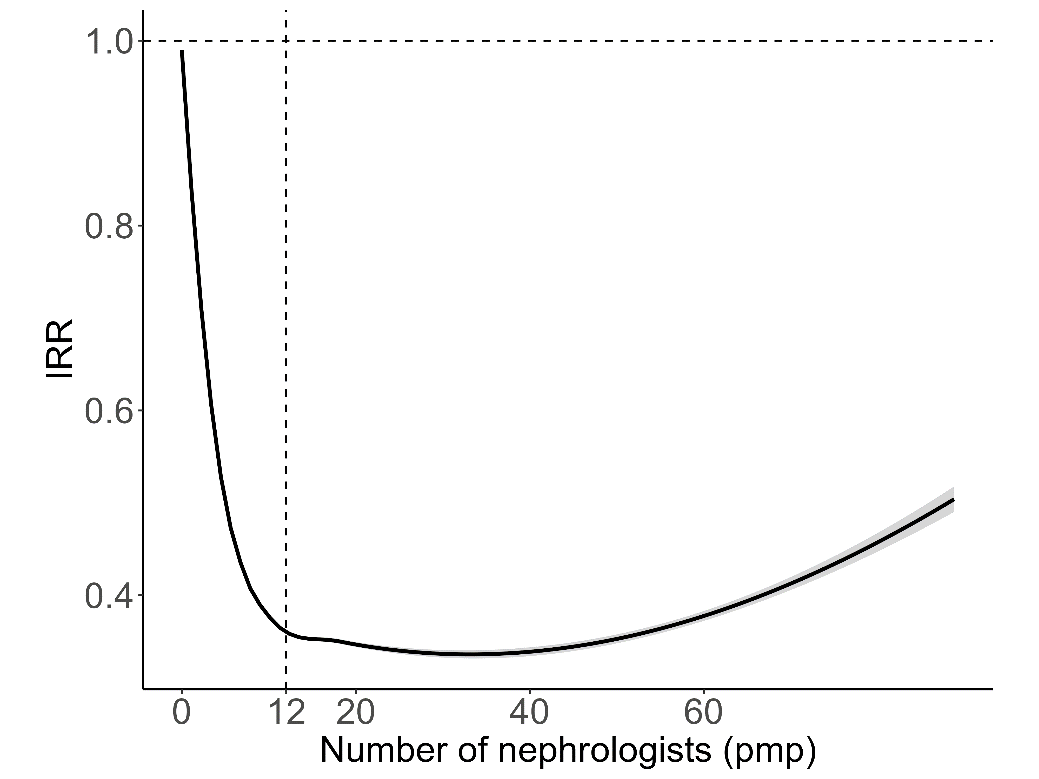


1. Number of nephrologists in the destination city


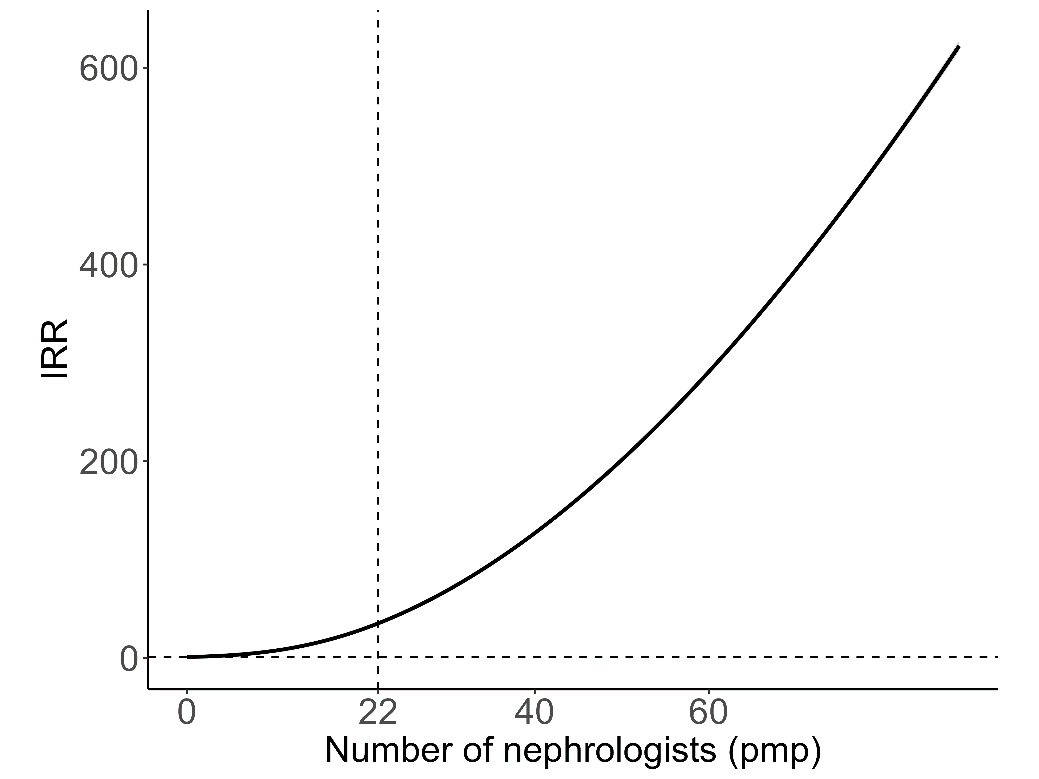


1. Proportion of nephrologists among physicians in the source city


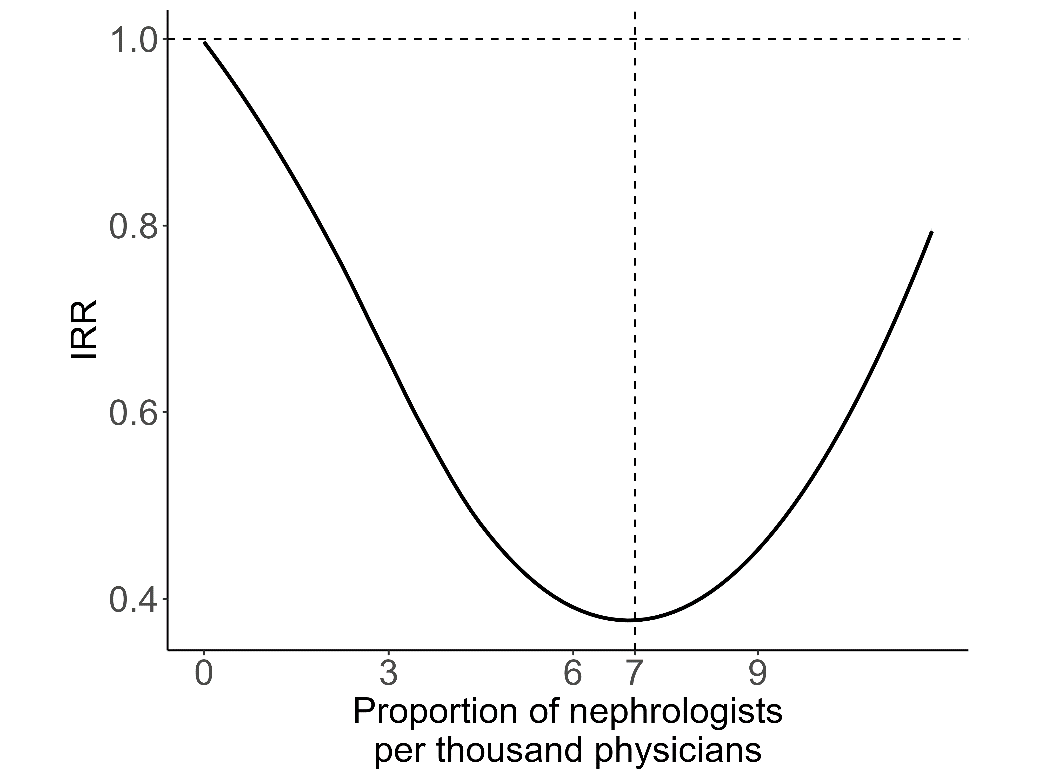


1. Proportion of nephrologists among physicians in the destination city


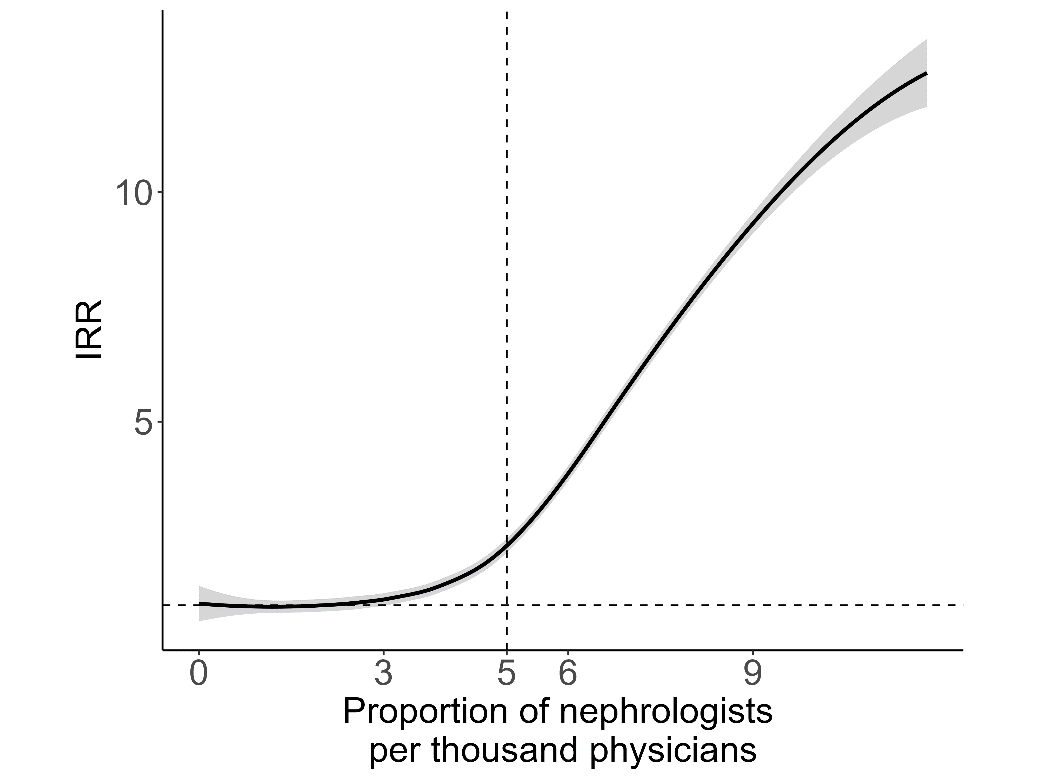


Figure S2. Non-linear effect of nephrology workforce on patient mobility for CKD in sensitivity analysis.
